# Newer Diabetes Management Options and Physical Fitness to Promote Cardiovascular Benefits

**DOI:** 10.31083/j.rcm2308282

**Published:** 2022-08-10

**Authors:** Eric Nylén

**Affiliations:** ^1^Veterans Affairs Medical Center, George Washington University School of Medicine, Washington, DC 20422, USA

**Keywords:** cardiovascular disease, cardiorespiratory fitness, diabetes medications, SGLT2 inhibitors, GLP-1 agonists

## Abstract

A plethora of diabetes studies and established clinical guidelines show the strong salutary benefit of aerobic, resistance, and/or combination exercise for improved glycemic and cardiovascular outcomes. Promotion of physical fitness is a cornerstone approach to improved diabetes management especially since subjects with diabetes have reduced baseline aerobic exercise capacity (i.e., reduced cardiorespiratory fitness) with associated increased risk for premature all-cause and cardiovascular mortality. Since medications are often used in conjunction with fitness promotion this can result in complex interaction between management modalities. More recently, newer options such as glucose transporter-2 inhibitors and incretin agonists have shown to improve cardiovascular disease (CVD) outcomes in cardiovascular outcomes trials. Indeed, both classes of agents have experimentally the potential to synergize with exercise training but clinical data vis-à-vis cardiorespiratory fitness is still preliminary. Review of the interaction of exercise and metformin shows no improvement in cardiorespiratory fitness. The use of glucose transporter-2 inhibitors may improve fitness performance in those with diabetes and heart failure. Although incretin agonists have physiological effects on the vasculature and heart, they lack similar clinical supportive data.

## 1. Introduction

Approximately 11% of the US population have diabetes of which 90–95% have type 2 diabetes (DM2) [1]. Atherosclerotic improve cardiovascular disease (CVD) is the leading cause of morbidity and mortality among individuals with diabetes and is the major cause of disability, reduced quality of life and premature death [2, 3]. Among the many treatment modalities, lifestyle promotion involving physical activity (PA) is a cornerstone approach both in terms of diabetes prevention as well as diabetes morbidity and mortality intervention [4, 5]. Indeed, it is widely known that cardiorespiratory fitness (CRF) and/or PA status among diabetes subjects is the strongest predictor of mortality [6, 7], yet successful and sustainable enhanced CRF is most often not achieved. Indeed, objective data using accelerometers substantiate the lack of PA adherence as only 5% of US adults reach more than 30 min of moderate intensity PA per day [8]. In a secondary analysis of the Look Action for Health in Diabetes (AHEAD) study, only those with sustained and improved effort captured by accelerometry (and not self-reported activity) showed salutary CVD outcomes [9]. Of considerable concern is that subjects with diabetes are both significantly sedentary and show reduced measured CRF [10, 11, 12]. Moreover, physical inactivity (PI) (not reaching the recommended level of PA and being sedentary) have been associated with cardiometabolic diseases such as diabetes, even in the presence of regular exercise [13]. Indeed, very low levels of occupation-related PA have been documented [14]. Physiological barriers include insulin resistance, vasculopathy, diminished oxygenation to exercising muscle, and dysfunctional mitochondria. Barriers more specific to type 1 diabetes (DM1) patients include fear of hypoglycemia while sociodemographic factors impact DM2 patients [15, 16]. Thus, it will be difficult to achieve the PA goals most recently set forth in the 2020 World Health Organization recommendation of 150–300 min of moderate intensity PA per week or 75–150 min of vigorous intensity PA per week or an equivalent combination of moderate intensity and vigorous intensity PA per week which was most recently reaffirmed in the 2022 consensus rendition of the American College of Sports Medicine for the majority of subjects [17, 18].

While CRF is a long-established independent predictor of CVD and overall mortality among subjects with diabetes and promotion of PA and higher CRF confer cardiometabolic benefits in proportion to the level of fitness (independent of body mass index (BMI)), the recent introduction of sodium glucose transporter-2 inhibitors (SGLT2i) and incretin modifiers such as glucagon like peptide-1 agonists (GLP-1a) in DM2 treatment has already impacted diabetes management significantly. Considering that CRF favorably modulates the adverse glycemic impact of statins [19], systematic scrutiny of possible medication interaction with CRF is warranted.

## 2. Metformin and Physical Activity 

**Fig. 1.**
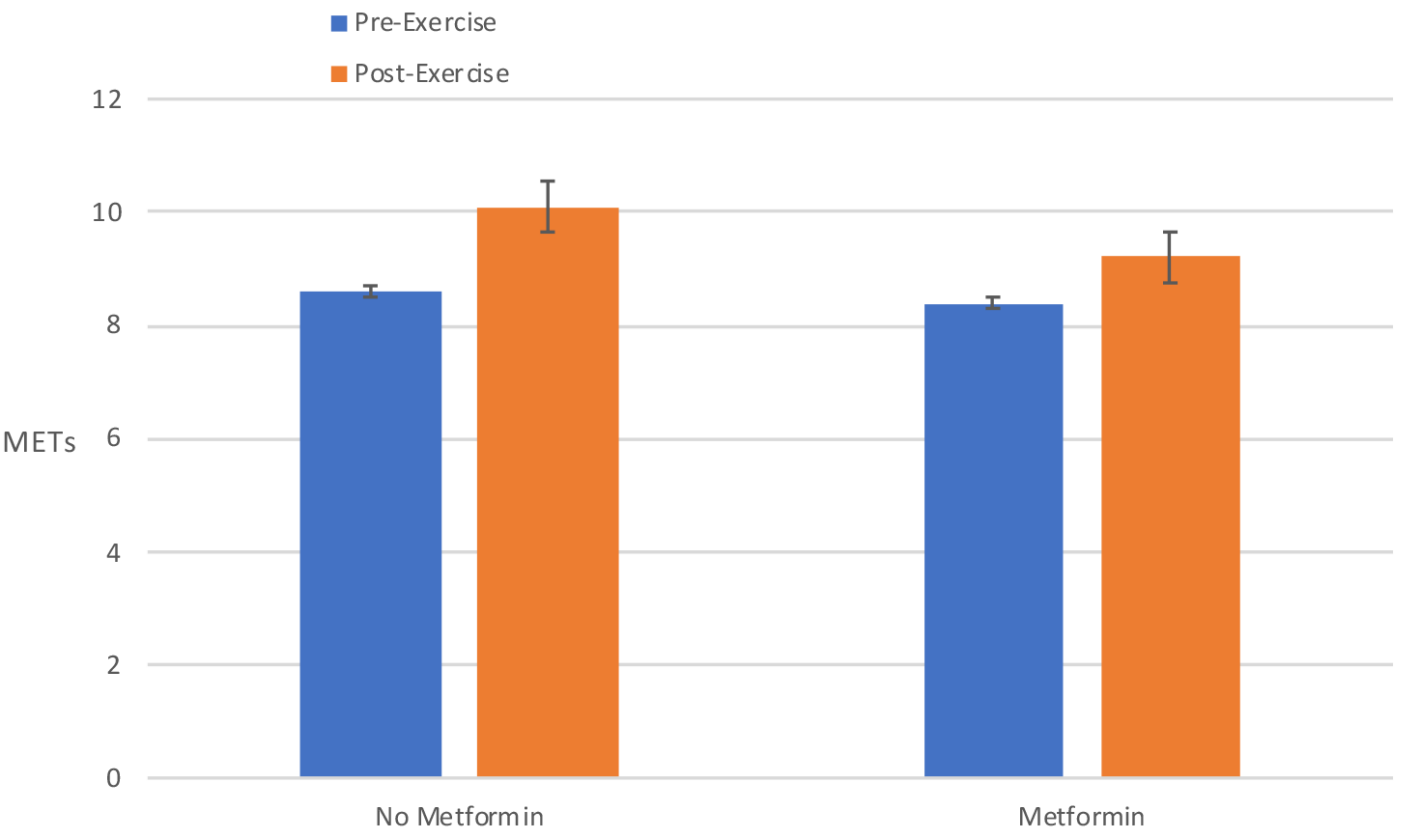
**Baseline and post-exercise cardiorespiratory fitness in peak metabolic equivalents (the Bruce protocol) following 12 weeks of combined aerobic and resistance exercise program in 147 type 2 diabetes subjects (n = 76 not on metformin; n = 71 on metformin)**. Metformin significantly attenuated post-exercise CRF which increased by 19% in those not on metformin and 12% in those on metformin (*p *< 0.005; ANOVA with repeated measures to test for changes between the two groups. Adjusted for age, gender, race, smoking status, beta-blocker and statin use).

Metformin is the most commonly prescribed diabetes medication and highlights the interaction with fitness promotion [20]. Metformin has multiple actions impacting glucose homeostasis; it acts on the liver to lower glucose production; it acts on the intestines to increase glucose utilization; it promotes the release of GLP-1 and GIP (via gut microbiome); and it lowers proinflammatory cytokines [21]. Metformin has effects on energy by inhibiting the mitochondrial respiratory chain-complex 1 with decreased adenosine triphosphate (ATP) production [21]. As with exercise alone, Metformin also activates AMP-activated protein kinase (AMPK) which result in improved insulin sensitivity [21]. At the level of skeletal muscle, mitochondrial respiration is decreased and lactate levels are higher [21]. The modifying potential of Metformin include elevated heart rate and lactate during exercise, increased perceived exertion, decreased glycemic response and hyperglucagonemia [22]. However, a follow up long-term study of a larger study reported no change in CRF results using metformin [23]. More acute studies also suggested that combination of metformin and exercise blunted the expected response [24]. Other studies in healthy and insulin resistance subjects similarly show modifying impact on metformin on CRF. Moreover, metformin blunted the hypertrophic response to resistance training (in older non-diabetic adults) [25]. As seen in Fig. 1, among predominantly African American males (80%) with diabetes for >10 years with an average BMI of 33.1 ± 5.0, a 12 week supervised combined aerobic and resistance program, the achieved metabolic equivalent for task (METs) was attenuated significantly in the metformin group (19% vs 12%, respectively; *p *< 0.005). Thus, there is some continued concern that the combination of Metformin with exercise may unfavorably impact important parameters such as CRF that impact cardiovascular outcomes.

## 3. Newer Diabetes Agents

SGLT2i are novel agents that act independently via non-beta cell renal glucosuria [26] and GLP-1a potentiate the incretin hormonal response to enhance glucose-dependent insulin secretion and inhibit glucagon secretion [27]. Fortuitously both SGLT2i and GLP-1a have shown improvement in CVD outcomes by reducing major adverse cardiovascular events (MACE) in randomized trials [28]. Although both pharmaceuticals are now vigorously promoted in diabetes management, very little is known about the interaction with fitness promotion, which is especially important in view of prior studies suggesting an attenuation with the combination of medications such as statins and metformin and exercise performance [19, 29]. Moreover, the mechanism(s) underlying the favorable CVD outcomes with these new agents are still being investigated [30, 31].

## 4. SGLT2i and Physical Activity

The mechanism of action of SGLT2i include a decrease in glucose reabsorption at the proximal renal tubules thereby increasing urinary glucose excretion with modest improvement in glycemic control. SGLT2i also modulates the intraglomerular pressure and has cardio-renal protection properties. Metabolically, SGLT2i use results in weight loss, lower blood pressure, and mild ketogenesis with glucagonemia. Multiple cardiovascular outcomes trials (CVOT) trials in DM2 have established that SGLT2i’s improve CVD outcomes [28]. For example, in the EMPA-REG OUTCOME study, Empagliflozin treatment of DM2 and CVD resulted in reduced MACE, death and hospitalization for heart failure [32]. In the CANVAS trials (CANVAS-R programs), Canagliflozin treatment of DM2 with high CV risk also reduced MACE, although CV death or overall mortality did not change [33]. Clearly, SGLT2i have a role in congestive heart failure (CHF) showing reduced incidence of both CHF and CHF-hospitalizations in patients with DM2 in those with underlying CVD. For example, Dapagliflozin reduced hospitalization for CHF both in patients with and without impaired left ventricular systolic function [34]. As shown in the landmark HF-ACTION randomized controlled trial, the addition of exercise resulted in significant reductions for both all-cause mortality and hospitalization and cardiovascular mortality which was dose related [35]. Since exercise intolerance is very much part of CHF, improvements in CRF have been associated with long-term reduction of clinical events. The effects of SGLT2i on CRF in patients with established CHF is being explored with mixed results so far; in the CANA-HF prospective randomized controlled study in patients with reduced ejection fraction and DM2, Canagliflozin did not improve measured CRF when compared to an incretin enhancer (sitagliptin) albeit the trial was terminated early [36]. In an open-label single arm prospective pilot study of Empagliflozin, CRF did not appear to improve in those with reduced ejection fraction unless it was used in conjunction with loop diuretics [37]. On the other hand, Empagliflozin in an uncontrolled pilot study was associated with 11% improvement in CRF after 1 month in DM2 patients with symptomatic CHF [38]. Similarly, treatment with Empagliflozin led to an increased CRF (i.e., peak oxygen consumption), and improvement in heart rate recovery [39]. In non-DM2 subjects with obesity, using a randomized, double-blind design, Dapagliflozin for 12 weeks with supervised endurance exercise training, the SGLT2i blunted insulin sensitivity independent of effects on aerobic fitness or body composition [40].

The interaction of SGLT2i and exercise is of interest related to the type of metabolic substrate used by the heart. With or without diabetes, SGLT2i accelerates lipolysis and fat oxidation with increased ketogenesis, especially that of β-hydroxybutyrate. In the diabetic heart, there is a mismatch between the uptake and oxidation of the abnormally elevated exogenous fatty acids which can decrease cardiac function termed “cardiac lipotoxicity” [41]. In experimental heart failure for example, exercise endurance was improved using Empagliflozin [42].

## 5. GLP-1a and Physical Activity

Glucagon Like Peptide-1 is secreted by the L cells of the distal small intestine and colon and has multiple physiological effects which include stimulating pancreatic insulin secretion (i.e., the incretin effect), suppressing glucagon secretion, suppressing appetite, slowing gastric emptying, and enhance thermogenesis with beneficial effects on energy balance. GLP-1 itself have beneficial effects on the heart with increased myocardial glucose uptake,vasodilation,and activation of cyclic adenosine monophosphate promoting cardiovascular protection [43]. Relevant to exercise, experimentally GLP-1 appears to reduce insulin resistance in skeletal muscle and improve endothelial and cardiac function [44, 45]. The CVD outcome trials using GLP-1a such as the LEADER trial showed reduced risk of MACE [46], while in the SUSTAIN-6 trial the rate of CVD death and non-fatal MI and stroke in patients with high cardiovascular risk was reduced [47]. Impaired GLP-1 secretion and thus impaired incretin effect is significantly diminished in diabetes, suggesting that altered GLP-1 secretion and/or function is associated with the underlying pathophysiology. The evoked response to exercise of endogenous GLP-1 may provide additional oxygen delivery to muscle via endothelial nitric oxide synthase activation in DM2 [48]. The use of once weekly albiglutide (in non-DM CHF subjects) appeared to improve CRF but of uncertain significance [49]. Moreover, Exenatide exposure for 3 months did not improve CRF [50]. The use of Liraglutide while improving weight and lipids, did not improve CRF or parameters of cardiac function [51]. Sitagliptin, an incretin agonist via a dipeptidyl peptidase-4 inhibition, did not improve CRF in DM2 subjects. Despite these negative findings, if GLP-1 action is blocked experimentally, the CRF and bioenergetics of the heart decreases [48].

## 6. Novel and Future Aspects of Physical Fitness and Diabetes 

In addition to the CRF modifying potential of medications. it has been recognized for some time that there is considerable pathophysiological heterogeneity among diabetes subjects. A novel cluster-based classification system divided diabetes into five subgroups based on disease progression and risk of diabetic complications [52]. Focusing on early-stage diagnosis of diabetes and fitness it was noted that in the severe insulin-resistant diabetes cluster (SIRD), who have increased nephropathy and non alcoholic fatty liver disease (NAFLD) and associated CVD risk, also showed the lowest baseline fitness and would be a preferential target with particularly favorable response to efforts to increase CRF [53].

Another advance is to better understand the complex molecular signaling with CRF that translates into health benefits, documentation of novel PA specific metabolites such as elevated choline plasmalogens and decreased ceramide, both implicated in diabetes pathophysiology, could serve as targeted biomarkers to enhance personalized exercise programs [54]. Moreover, the so-called exerkines is a group of signaling substances associated with muscle contraction which is expanding beyond the originally established role of IL-6 release which could also be useful in expanding the basic concepts of exercise and interactions with medications [55].

## 7. Summary

Among diabetes subjects, CRF is an important modifiable cardiovascular risk factor while also an underutilized vital sign which in diabetes is pathophysiologically linked to insulin resistance, mitochondrial dysfunction, vascular and cardiac abnormalities [56]. Sustaining enhanced fitness and CRF in subjects with diabetes may be more impactful than the challenges of obesity and dietary adherence [57, 58]. Importantly, achieving even very modest improvement in CRF is highly significant for cardiovascular outcomes among those with diabetes including those with obesity [59]. Since fitness promotion is often associated with concurrent use of diabetes medications, some of which has in some studies been reported to attenuate CRF, it is important to more thoroughly examine the interaction to optimize outcomes such as CVD.

## Abbreviations

AMPK, AMP-Activated Protein Kinase; BMI, Body Mass Index; CHF, Congestive Heart Failure; CRF, CardioRespiratory Fitness; CVD, CardioVascular Disease; EMPA-REG OUTCOME, CardioVascular Outcomes Trials; DM1, Type 1 Diabetes; DM2, Type 2 Diabetes; GIP, Glucose Inhibitory Peptide; GLP-1a, Glucagon Like Peptide-1 Agonist; IL-6, InterLeukin-6; MACE, Major Adverse Cardiovascular Events; MET, Metabolic Equivalents of Task; NAFLD, Non Alcoholic Fatty Liver Disease; PA, Physical Activity; SGLT2i, Sodium Glucose Transporter-2 Inhibitors.
